# Nano-Based Drug Delivery and Targeting to Overcome Drug Resistance of Ovarian Cancers

**DOI:** 10.3390/cancers13215480

**Published:** 2021-10-31

**Authors:** Melayshia McFadden, Santosh Kumar Singh, Gabriela Oprea-Ilies, Rajesh Singh

**Affiliations:** 1 Department of Microbiology, Biochemistry and Immunology, Morehouse School of Medicine, Atlanta, GA 30310, USA; mmcfadden@msm.edu (M.M.); sksingh@msm.edu (S.K.S.); 2 Department of Pathology & Laboratory Medicine, Winship Cancer Institute, Emory University School of Medicine, Atlanta, GA 30322, USA; goprea@emory.edu; 3 Cancer Health Equity Institute, Morehouse School of Medicine, Atlanta, GA 30310, USA

**Keywords:** ovarian cancer, nanoparticles, targeted drug delivery, drug resistance

## Abstract

**Simple Summary:**

Ovarian cancer (OvCa) is a prominent cause of cancer death in women due to missed early signs and late diagnoses. Once a woman is diagnosed with OvCa, the standard treatment is surgery to remove the tumor, followed by chemotherapy. Many women go into remission after treatment, but there is always a strong possibility that the cancer will return. If the cancer returns in less than 6 months, the patient is considered platinum-resistant and undergoes a new treatment plan. Drug resistance occurs when the cancer cells become resistant to the administered drug during initial chemotherapy, causing the drug to become ineffective. This is a considerable challenge in the cancer field, and many researchers are looking for strategies to overcome this drug resistance. However, nanotechnology, natural products, and RNA interference therapy are strategies that can enhance cancer therapy to overcome drug resistance in cancer cells.

**Abstract:**

Ovarian cancer (OvCa) is a destructive malignancy due to difficulties in early detection and late advanced-stage diagnoses, leading to high morbidity and mortality rates for women. Currently, the quality treatment for OvCa includes tumor debulking surgery and intravenous platinum-based chemotherapy. However, numerous patients either succumb to the disease or undergo relapse due to drug resistance, such as to platinum drugs. There are several mechanisms that cause cancer cells’ resistance to chemotherapy, such as inactivation of the drug, alteration of the drug targets, enhancement of DNA repair of drug-induced damage, and multidrug resistance (MDR). Some targeted therapies, such as nanoparticles, and some non-targeted therapies, such as natural products, reverse MDR. Nanoparticle targeting can lead to the reversal of MDR by allowing direct access for agents to specific tumor sites. Natural products have many anti-cancer properties that adversely regulate the factors contributing to MDR. The present review displays the current problems in OvCa treatments that lead to resistance and proposes using nanotechnology and natural products to overcome drug resistance.

## 1. Introduction

In the developed world, ovarian cancer (OvCa) is one of the leading causes of cancer deaths among women. In the United States, approximately 1 in 78 women will develop OvCa in their lifetime; it is mainly diagnosed in women aged 64 years or older [1,2]. High-grade serous ovarian cancers, which account for 68% of OvCa cases, are clinically aggressive neoplasms that develop from the ovarian surface’ epithelium. High-grade serous OvCa is usually diagnosed at a late and advanced stage and has the worst prognosis [3]. Therefore, screenings and early detection provide the opportunity for effective treatment. However, the symptoms associated with OvCa are often vague and may be dismissed because they are similar to the effects of the cyclic hormonal changes that occur naturally within the body [4,5]. Current treatment options include combinations of tumor debulking surgery, chemotherapy, and radiation therapy. Advanced treatment options include hormone therapy and targeted therapy [6]. Even with treatment, a substantial proportion of advanced OvCas will develop resistance within 18 months [7,8]. Mechanisms of drug resistance include drug inactivation, multi-drug resistance (MDR), alterations of drug targets, and enhanced DNA repair [9]. However, the most common mechanism of drug resistance in OvCa is the stimulation of ATP-dependent membrane efflux pumps, especially Multidrug resistance protein 1 (MDR1), also known as P-glycoprotein (P-gp) or ABCB1 [10,11,12,13,14,15]. Understanding these mechanisms can lead to advancements in the treatment of OvCa by using strategies like nanoparticles that can target and reverse MDR by allowing direct access of drugs to specific tumor sites. Furthermore, natural products can also reverse MDR due to their anti-cancer properties. In this review, we present the current problems of OvCa treatments, outline the various mechanisms involved in OvCa drug resistance, and propose the use of nanotechnology and natural products to overcome OvCa drug resistance.

## 2. Treatments of OvCa

### 2.1. Tumor Debulking Surgery

Once a patient is diagnosed with OvCa, they undergo tumor debulking surgery to determine the stage of the disease and the cancer prognosis [16]. The staging assessment by surgical pathologic degrees is accomplished by following the International Federation of Gynecology and Obstetrics (FIGO), where a 5-year survival rate between 90% and 10% is determined based on stages I, II, III, and IV [17,18]. There are three broad classifications of prognostic factors for OvCa: the tumor, the patient, and the clinical interventions [19]. According to the Gynecologic Oncology Group, optimal cytoreduction is defined as a residual tumor of less than 1 cm after surgery and suboptimal cytoreduction as resulting in any larger residual tumor [20,21,22]. Such measurements are subjectively determined after surgery. If optimal debulking is achievable, it is preferable to perform primary surgery, but primary surgery should be avoided if there is a probability of suboptimal debulking [23,24,25,26,27]. For advanced stages of cancer (III and IV), complete cytoreduction is often not possible. Patients who are too ill or have inoperable lesions are treated with three cycles of neoadjuvant chemotherapy. If there is a response to the chemotherapy, an interval debulking surgery will be conducted, followed by six cycles of chemotherapy [28]. When completing tumor debulking surgery, the ultimate goal is the resection of all residual diseases, whether performed primarily or secondarily. However, recurrence occurs in 75% of patients despite an initial response. The remaining objective is to find an alternative approach to treat OvCa.

### 2.2. Chemotherapy

After surgery, chemotherapeutic drugs are used to treat patients. The most common agents for the treatment of OvCa are carboplatin and cisplatin [29]. In the early 1980s, carboplatin was introduced as an equivalent of cisplatin due to its similarities in response rate and survival outcomes. However, carboplatin is preferred over cisplatin due to the potential for nephrotoxicity, ototoxicity, and nausea or vomiting seen with cisplatin [30,31,32,33,34]. Carboplatin is an alkylating agent that inserts platinum into DNA to form crosslinks. The resulting structural distortion of the DNA triggers a signaling cascade resulting in apoptosis [35,36]. Patients who relapse within 6 months of platinum treatment are considered drug-resistant, and they have a median survival time of less than a year [37]. However, the standard treatment of platinum-resistant OvCa is non-platinum single-agent chemotherapy, such as paclitaxel (PTX). In the 1990s, PTX was the most efficient chemotherapeutic agent for patients with relapsed platinum-resistant OvCa [38]. PTX is cell cycle-specific and binds to β-tubulin, which causes microtubule stabilization, G_2_-M arrest, and apoptosis. However, resistant cancer cells treated with PTX remain in mitosis until the drug clears and then continue proliferation, resulting in PTX resistance [39,40]. Currently, it is not possible to predict a patient’s response to chemotherapy, and it is unlikely for a patient to advance from further chemotherapy after failing two lines of treatment due to the inevitable toxicities and side effects associated with treatment.

### 2.3. PARPi Frontline Therapy for Ovarian Cancer

Decades of research have investigated drug resistance and targeted therapy for OvCa patients. After initial surgery and chemotherapy, there is often no evidence of disease; however, most patients experience recurrence [41]. In light of such resurgence following initial therapy and understanding and deciphering the situation at the molecular level, poly-ADP-ribose polymerase (PARP) inhibitors have been developed. A recent report established that olaparib, niraparib, or rucaparib [41,42] reduced DNA repair in cancers with a BRCA gene mutation, leading to cancer cell death. Since empirical findings support an anti-tumorigenic role, several clinical trials have been conducted that display promising progression-free survival (PFS) (NCT04573933). According to the phase two trial of rucaparib, patients with platinum-sensitive, high-grade ovarian carcinoma with a BRCA mutation (germline or somatic) and high chromosomal loss of heterozygosity have increased PFS [43]. The FDA has approved this drug for treating advanced OvCa. Another breakthrough FDA approval is for olaparib monotherapy, given to OvCa patients who received chemotherapy and have a suspected germline BRCA mutation. Furthermore, the European Medicines Agency (EMA) approved olaparib for high-grade serous epithelial ovarian, fallopian tube, or primary peritoneal cancer with a germline or somatic mutation that responds to platinum therapy [43]. Moreover, scarcity of tumor suppressor genes such as ATR, ATM, PALB2 [43], and PTEN [44] also confers susceptibility to PARP inhibitors. Growing evidence suggests that the loss of PTEN increases chromosomal instability [45] and replication fork collapse [46] in most cancers. In contrast, both BRCA mutant and BRCAness cancers are resistant to PARPi therapy [43,46], which leaves the unanswered question of how cancer cells present defensive measures.

### 2.4. Chimeric Antigen Receptor-Modified T (CAR-T) Cell Therapy

Despite the initial response to chemotherapy, OvCas generally recur, and most patients have low median survival rates [47]. In this context, an effective therapeutic approach is urgently required to achieve the long-term survival of OvCa patients [48]. The most recent clinical approach for cancer treatment includes adoptive T cell transfer (ACT). In this therapy, the patient’s T cells and natural killer cells are isolated, modified, and expanded ex vivo to achieve target immune response and eliminate cancer cells. Based on the current literature, the ACT is classified into three forms: tumor-infiltrating lymphocytes (TILs), T cell receptor (TCR)-T cells, and chimeric antigen receptor (CAR)-T cells [49]. The use of CAR-T engineered T cells is a promising treatment for OvCa [50] and hematological tumors [51,52]; however, its limitations are side effects and related toxicities. Methods to construct CARs include viral and non-viral transduction [48]. Cumulative evidence suggests that non-viral strategies such as liposomal-mediated, mRNA-mediated, and transposase system-mediated gene transfer have a lower risk of complication and are easier to produce than viral approaches [47]. Targeting solid tumors is more challenging due to their histopathological features, extensive vascular leakage, T-cell trafficking, and infiltration into tumor sites. This impairment occurs due to an enhanced permeability and retention (EPR) effect, heterogeneity [53], aberrant vasculature [54], and a hypoxic and immunosuppressive tumor microenvironment [55]. Nevertheless, various CAR-T biological effects are implicated in cancers, including OvCa [48,56]. Clinical trials conducted for CAR-T therapy of OvCa are mainly in the early phases. Furthermore, the targeting of various antigens also has excellent potential in treatment. CAR-T has a high affinity for cell surface antigens; however, T-cell antigen receptor (TCR)-T recognizes the intracellular and surface antigens presented by major histocompatibility complex (MHC) molecules [57]. Thus, CAR-T cell therapy could be developed as an alternative treatment for OvCa.

## 3. Mechanisms of Drug Resistance

**Figure 1:**
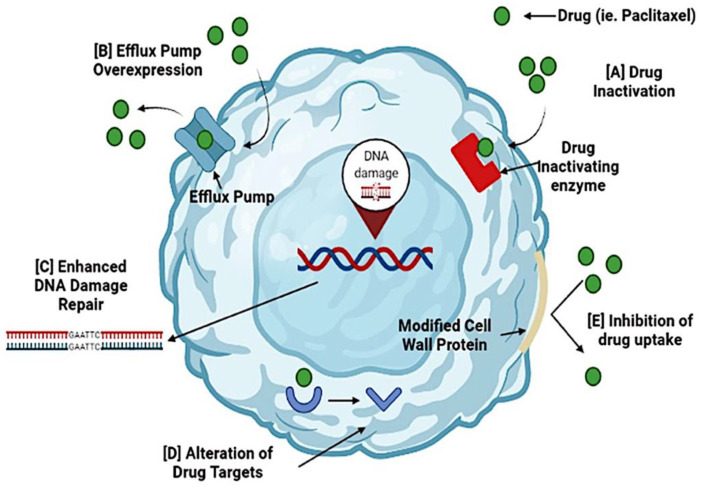
Mechanisms of drug resistance in ovarian cancer. Cancer cells can become resistant to the administered drug during chemotherapy. Several mechanisms cause resistance to chemotherapy. (A) Activation of a drug occurs when resistance genes (MDR) code for enzymes that chemically modify the drug. (B) Efflux pump overexpression inhibits the accumulation of a chemotherapeutic drug. (C) Enhanced DNA damage repair is triggered by DNA repair mechanisms, nucleotide excision repair, and homologous recombination. These processes reverse drug damage and increase drug resistance. (D) Alteration of drug targets occurs because chemotherapeutic drugs have specific targets, and changes may render the drug ineffective. (E) Inhibition of drug uptake occurs due to modifications in the cell wall proteins, preventing drugs from entering the cell. By targeting these resistance mechanisms, cancer cells can become sensitive to the drugs and increase their effectiveness.

The drug resistance of tumor cells develops due to various factors, including the inactivation and alteration of drugs, DNA repair augmentation, and multidrug resistance (MDR) [9]. A schematic presentation of the MDR mechanism is shown in Figure 1.

### 3.1. Drug Inactivation

For some drugs to have clinical efficacy, they must avoid metabolism. Advanced OvCas often develop resistance to platinum treatment via drug inactivation. This occurs when metallothionein and the thiol glutathione switch on a detoxification system and reduce the damage to cancer cells [58,59]. Furthermore, the tumor suppressor gene p53 is involved in drug inactivation. The gene p53 has the potential to maintain the homeostasis and genomic integrity of the target genes. Gene p53 participates in many cellular processes such as activation of cell cycle arrest, apoptosis, and DNA repair mechanisms in cancers, including OvCa [60]. However, if p53 is mutated or deleted, apoptosis is inhibited, and drug resistance occurs [61]. Drug resistance occurs because p53 acts as a homotetrameric transcriptional factor, and its mutations lead to three different phenotypes: loss of function (LOF), dominant negative (DN), and gain of function (GOF). LOF is the leading result from mutations and simply means that there is a loss of the functions that the wild-type (WT) p53 possessed. Missense mutations of p53 can cause a DN effect, causing WT p53 to lose transcriptional activity or gain novel oncogenic functions or GOF mutants. This GOF effect has been seen quite often in previous studies that demonstrated the transcriptional dependence between ABCB1 and p53 [62]. Overall, mutated p53 will disable cells from defending themselves against carcinogenesis and promote cancer cells to undergo proliferation, drug resistance, and metastasis [61,63].

### 3.2. Alteration of Drug Targets

PTX resistance can occur in OvCas due to alterations of drug targets (e.g., a mutation in β-tubulin). Microtubules are created by α- and β-tubulin heterodimers associating together in a head-to-tail fashion. PTX binds to β-tubulin, leading to microtubule stability and polymerization. Thus, blocking the cell cycle, especially the mitosis phase, induces apoptosis [64,65]. However, β-tubulin mutations cause the opposite effect in microtubule dynamics and stability, erode the interactions between PTX and β-tubulin, and increase resistance to PTX [66,67,68,69,70,71].

Along with the alteration of drug targets, drug resistance can occur through altering the process of drug activation. The resistance mechanism is thought to be associated with the loss of phosphatase and tensin homolog (PTEN) function, cell cycle inhibition, co-expression of growth factor receptors, and upregulation of the PI3K/Akt pathway [59,72,73]. Overexpression of the PI3K/Akt pathway downregulates PTEN expression and increases resistance to PTX [74,75,76].

### 3.3. DNA Damage Repair

Chemotherapeutic agents may directly or indirectly damage the DNA of cancer cells. However, cancer cells can recognize the damaged DNA and repair it [9]. For example, for many years, treating OvCa was solely performed by using chemotherapy with platinum-based drugs like carboplatin, which causes DNA crosslinks, leading to apoptosis and cell death. However, prolonged exposure of OvCa to platinum drugs leads to resistance due to nucleotide excision repair, DNA repair mechanisms, and homologous recombination [9,59,77]. In OvCas, these DNA repair systems are primarily involved in reversing platinum damage and increasing drug resistance. Nevertheless, inhibition of the DNA repair systems sensitizes cancer cells to these drugs, thus increasing the potency of chemotherapy. Therefore, in cancer cells, the efficacy of these chemotherapeutic agents is dependent on the inhibition of the DNA repair systems.

### 3.4. Multidrug Resistance

A challenge in cancer treatment is multidrug resistance (MDR), which provides cancer cells with the capacity to survive against exposure to an extended range of anti-cancer drugs [78]. MDR can develop by several mechanisms, including decreased drug uptake, increased detoxification, or increased efflux by upregulation of ATP-binding cassette (ABC) transporter genes [39,79]. The MDR to chemotherapeutic drugs in cancer cells is mediated through a mechanism involving P-glycoprotein (P-gp, or MDR1), multidrug resistance-associated protein 1 (MRP1, or ABCC1), or breast cancer resistance protein (BCRP, or ABCG2) [9,80]. P-gp, which is required for the normal passage of chloride ions in and out of the cell, binds to chemotherapeutic drugs such as PTX. This binding results in ATP hydrolysis at the nucleotide binding site, leading to a change in configuration and to efflux out of the cell, thus rendering them inactive [10,11,12,81]. Another class of efflux pumps that mediates MDR in cancer cells toward taxanes is the MRP family, referred to as adenosine triphosphate-binding cassette C group (ABCC) transporter proteins [39,77,82]. One such member is MRP1, an ABC transporter that effluxes drugs and organic anions across the plasma membrane. In the absence of MDR1 or P-gp expression, MRP1 provides drug resistance for various types of cancer. Another carrier, breast cancer resistant protein, which is coded by ABCG2, is overexpressed in OvCas [83]. Moreover, extracellular vesicles (EVs) are of growing interest as modulators of drug resistance within the tumor microenvironment. These act as cargo carriers between tumor cells and stromal cells. EVs are small in size (30–150 nm), involved in metastasis, and can direct the function of a cell by carrying nucleic acids, proteins, or lipids [84]. We may overlook various facets of EVs that result in the modulation of drug resistance. Based on recent studies, identifying a vesicular component, such as miR21, which contributes to taxane resistance, might be helpful to develop a therapeutic option for OvCa by interfering with drug uptake [85]. Overall, these classes of efflux pumps, alone or in combination, reduce the cancer cells’ sensitivity to the chemotherapeutic drugs, leading to the failure of the OvCa treatment. Therefore, to overcome these challenges, strategies to use monoclonal antibodies or chemical compounds that bind to and render inactive these efflux pumps have been developed. Secondly, drug combinations were prepared to allow higher doses of chemotherapy drugs, and alterations to the structures of chemotherapy drugs were made. The objective was to make the drug incapable of binding to the efflux proteins. Finally, strategies have been used to inactivate the MDR genes at the transcriptional level by targeting their mRNAs [86]. These strategies often involve the use of natural products or nanotechnology.

### 3.5. Overcoming Resistance to the Checkpoint Blockade

The elimination of cancer cells through the immune system has been a dream for cancer biologists. Traditional methods to reduce cancer stimulate the immune system with vaccines, dendrite cell activation, or immunostimulants. Despite the use of this defense mechanism, tumor cell elimination remains a challenge. The cancer cells escape immunity through loss of tumor cell recognition by T-cells, creation of an immunosuppressive micro-environment, or modulation of the immune checkpoint [63]. A critical element of the checkpoint mechanism is represented by the programmed cell death protein 1 (PD-1) and programmed cell death-ligand 1 (PD-L1) interaction, which results in T-cell deactivation, preventing healthy cells from being targets of T-cell-toxicity [63]. Among the several strategies to tackle cancer cells through the immune system is to use the checkpoint inhibitors that target the immune escape mechanisms and restore the T-cells of the host to total anti-tumor activity. Although the blockade of PD-1/PD-L1 requires reactivation and clonal proliferation of the T-cells present in the tumor microenvironment, failure of the checkpoint inhibitors may lead to (1) insufficient generation of anti-tumor T cells, (2) inadequate function of tumor-specific T cells [66,67], or (3) impaired formation of T-cell memory [64,68]. Approved checkpoint inhibitors include nivolumab, pembrolizumab, and cemiplimab for PD-1; atezolizumab, durvalumab, and avelumab for PD-L1; and ipilimumab for CTLA-4 [69]. Despite using these potent and broadly active inhibitors of PD-1/PD-L1, most patients do not respond or develop tumor progression or recurrence after a secondary response [70]. Clinical trials are underway to overcome the resistance toward checkpoint inhibitor therapy by evaluating a combination of checkpoint inhibitors along with targeted agents, cytotoxic chemotherapy, or radiation.

## 4. Natural Products as Modulators to Reverse MDR

In addition to current advanced technologies, natural products from various sources are being evaluated for cancer therapy. Natural products used for cancer therapies have low toxicity and are well-tolerated in the human body [87,88,89,90]. They exhibit anti-cancer activities via various mechanisms of action (Table 1). In this context, we provide information on several phytochemicals, including alkaloids, flavonoids, and terpenes, as modulators of MDR.

### 4.1. Alkaloids

#### Piperine

Piperine is a natural alkaloid isolated from the long and black pepper species *Piper longum* and *Piper nigrum*. Piperine possesses health properties, such as antioxidant, anti-inflammatory, antimicrobial, neuroprotective, and anti-cancer properties [91,96]. Against human OVACAR-3 cells, piperine induces cell cycle arrest in the G_2_/M phase, caspase activation, and cell migration inhibition of the PI3K/Akt/GSK3β signaling pathway [91]. Furthermore, piperine affects diverse signaling molecules and pathways associated with cancer cell growth and survival by targeting PI3K/Akt, MAPK, and STAT3 [97,98]. These results suggest that, for these cells, piperine exerts anti-cancer effects involving apoptosis induction and cell cycle arrest. Piperine is a promising agent to increase the sensitivity of cytotoxic drugs such as PTX and in targeting the drug resistance mechanisms in OvCa cells [99]. Based on clinical trial data, piperine delays prostate cancer progression, smoldering multiple myeloma (SMM), and monoclonal gammopathy of unknown significance (MGUS) when given in combination with curcumin (NCT04731844). Thus, piperine should be considered as a potential anti-cancer agent to improve the effectiveness of therapy for cancer patients.

### 4.2. Flavonoids

Flavonoids, including flavones, flavonols, isoflavones, flavanols, flavanonols, flavanones, and chalcones, are the most utilized and analyzed natural products in cancer research [100]. This is due to their capacity to reverse MDR by killing resistant cancer cells or becoming re-sensitized to anti-cancer drugs. The flavonoids fisetin, chrysin, quercetin, kaempferol, baicalein, rutin, and icariin, as well as iso-flavonoids, genistein, etc. and biochanin A reverse MDR by inhibiting the efflux effects of ABC transporters [101].

#### 4.2.1. Curcumin

Curcumin is a mixture of curcuminoids derived from the Indian spice turmeric [102]. Curcuminoids are notable for their anti-cancer, anti-inflammatory, antioxidant, and anti-viral properties [103,104,105,106,107]. Curcumin is commonly used to circumvent MDR to various anticancer agents [86]. By inhibiting the functions of ABC transporters P-gp, MRP1, and ABCG2, it restores drug sensitivity for various cancer cells [102,108,109,110,111]. Furthermore, in cisplatin-resistant OvCa cells, curcumin induces cell cycle arrest in the G_2_/M phase by increasing the apoptosis and phosphorylation of p53 [93]. However, curcumin is lipophilic and highly insoluble, so it has poor bioavailability and is efficient only in high doses. Rapid metabolism is a major problem encountered by using this and other natural products [108,112]. This natural product has minimal toxicity, as it and other flavonoids are used as dietary supplements. However, due to its lipophilic characteristics, it often requires a vehicle to assure its bioavailability.

Curcumin has been extensively studied in various models for a wide range of human diseases, including cancer. Animals receiving curcumin in combination therapy for cancer have longer median survival times [113,114]. For patients who relapse after surgery, curcumin prevents prostate cancer cell proliferation by blocking the enzymes needed for cell growth (NCT02064673). Many clinical trials have been completed and are ongoing on its safety and efficacy. Indeed, 72 trials have been conducted with curcumin; some of the ongoing clinical trials on curcumin are in phase three (prostate cancer, NCT03769766) or phase one (breast cancer, NCT03980509). Thus, curcumin shows promise for preventing and treating various cancers, including OvCa.

#### 4.2.2. Resveratrol

Resveratrol (RES) is a polyphenol found in grapes, red wine, pines, peanuts, mulberries, and various products derived from other plant species. RES exerts anti- or pro-oxidant effects, binds to and modulates various molecular targets, inhibits tubulin polymerization, and induces apoptosis via cell cycle arrest at the G_2_-S checkpoint [115,116,117,118,119,120]. In a study of autophagy, RES-treated OVCAR-3 cells induced the generation of reactive oxygen species (ROS) and apoptosis [94]. RES has exceptional anti-cancer properties, and its pharmacokinetics, metabolism, and toxicity have been evaluated. RES has poor bioavailability, however, because it is metabolized quickly into glucuronide and sulfate conjugates, which are excreted through the urine [121]. However, present data suggest that RES is appropriate for cancer treatment and will be effective in combination with chemotherapy agents and targeted therapies.

RES has an anticancer effect on numerous cancer cells. However, the efficacy of this compound in animals is not promising, and results are inconsistent due to poor bioavailability in rodents and humans [122]. Studies suggest that many factors need to be considered, such as target the WNT [123] and silent information regulator (SIRT) signaling pathways [124], before being used for cancer therapy. RES modulates WNT signaling, which is activated in >85% of colon cancers (NCT00256334). In a clinical study, a combination of RES (1000 mg twice a day) and myo-inositol (1000 mg twice a day) was effective for women diagnosed with polycystic ovary syndrome (NCT04867252). Generally, RES is effective for cancer prevention if taken orally as a supplement.

### 4.3. Terpenes

#### Thymoquinone

Thymoquinone (TQ), a terpene, is the active ingredient of the volatile oil of *Nigella sativa*, commonly known as black cumin or black seed [125]. TQ has anti-cancer characteristics that activate tumor suppressor genes such as PTEN and p21, reduce pro-inflammatory and angiogenic signals, and decrease DNA damage by inhibiting ROS formation [126,127,128,129,130,131]. Furthermore, by modulating the resistance mechanisms, TQ sensitizes cancer cells to the standard treatments of chemotherapy and radiotherapy [132]. The combination of TQ and cisplatin leads to better results than when used separately, with a higher apoptosis rate and Bax/Bcl-2 ratio [95]. These results indicate that TQ may be an agent for development as an OvCa drug.

## 5. Nanocarriers as Vectors to Overcome MDR 

### 5.1. Nanoparticles

Nanotechnology applied to chemotherapeutic agents for cancer treatment can overcome drug resistance by inhibiting the function of various mechanisms, such as the efflux transporters on cell membranes. Nanoparticles provide a new way to deliver anti-cancer drugs by allowing direct access to the cells and providing a drug combination therapy platform [133]. The size, characteristics, and enhanced permeability and retention (EPR) effect for nanoparticle construction are the primary considerations [134]. For cancer therapy, nanoparticles with diameters of 10–100 nm achieve EPR and deliver drugs effectively. However, particles with sizes less than 1–2 nm can leak from the normal vasculature and damage normal cells. Particles larger than 100 nm can be cleared from circulation by phagocytes [134,135,136].

Moreover, surface modifications can impact the nanoparticle’s half-life and its bioavailability. Therefore, nanoparticles are commonly altered to become hydrophilic, which increases drug circulation times and enhances penetration and accumulation in tumors [134,137,138,139]. For drug delivery by nanocarriers, targeting cancer cells is an essential characteristic, as it enhances the therapeutic efficiency of chemotherapeutic drugs and protects normal cells from cytotoxicity [134]. However, for a nanoparticle to deliver the chemotherapeutic agent, it must target the cells either passively or actively. A wide range of studies highlights that passive targeting of nanoparticles acts through the EPR effect that exploits the high vascular permeability and weak lymphatic drainage of cancer cells. The interaction between ligands and receptors achieves active targeting of nanoparticles. The receptors found on cancer cells include transferrin receptors, folate receptors, glycoproteins, and epidermal growth factor receptors [134]. Once the nanoparticle reaches the cancer cell and binds to a receptor, the drug is released to induce apoptosis. Nanoparticles are generally encapsulated with chemotherapeutic drugs or nucleic acids, implying that they can be involved in cytotoxicity and gene therapy [140]. In addition, nanoparticles can be used to encapsulate poorly soluble drugs and deliver them into circulation [141,142].

### 5.2. Types of Nanoparticles

The major types of nanoparticles used for cancer therapy and overcoming MDR include polymeric and solid lipid nanoparticles, liposomes, micelles, mesoporous silica nanoparticles, dendrimers, nanostructured lipid carriers, RNA interference structures, and planetary ball-milled (PBM) nanoparticles (Table 2).

#### 5.2.1. Polymeric and Solid Lipid Nanoparticles

Polymeric nanoparticles (size: 3–200 nm), extensively used as drug carriers, are created by binding a copolymer to a polymer matrix. The nano-formulations include synthetic polymers such as poly(lactic-co-glycolic acid) and polycaprolactone (PCL) and natural polymers such as a polysaccharide or polypeptide [152,153]. The sizes of solid-lipid nanoparticles (SLNs) range from 50–1000 nm. They are round, colloidal particles made up of lipids, chemotherapeutic drugs, and surfactants. Since spherical or round-shaped nanoparticles are favored for drug delivery, SLNs generally have better efficiency and capacity to overcome MDR by increasing drug uptake into cancer cells and inducing apoptosis [86]. Clinical tests have been conducted to assess chemotherapeutic drugs, including PTX, doxorubicin, camptothecin, and platinates as drug conjugates for various cancers. For instance, a combination of PTX and carboplatin polymeric nanoparticles was compared to the free drugs in targeting SKOV-3 and HO-8910 cells. Encapsulation of the drugs into nanoparticles increased their potency for OvCa cells [143]. Treatment with folic acid-PEGylated calix [4] arene nanoparticles reduced the tumor volumes of SKOV-3 xenografts compared with the free drugs. A study for identifying the role of PTX-encapsulated SLNs [144] found similar cytotoxicity to commercial Cremophor EL-based PTX in MCF-7 (breast) and OVCAR-3 cells, suggesting that SLN-based nanoparticles are an effective delivery system for various routes of administration.

#### 5.2.2. Liposomes

Liposomes (size: 50–200 nm) are spherical and contain one or multiple layered membrane structures. Liposomes are stable, biocompatible, biodegradable, non-toxic, and non-immunogenic, and they can accumulate in tumor cells by the EPR effect [154]. Strategies to augment drug bioavailability and efficiency for drug-resistant cancers include liposomes adapted for controlled release and ligand-targeted drug delivery into tumor cells [155]. Currently, PTX-, vincristine-, and camptothecin-encapsulated liposomes are in clinical tests. For example, Qi et al. (2018) [145] prepared PTX encapsulated in PEGylated liposomal nanoparticles (PL-PTX) to improve the efficiency in suppressing and killing OvCa cancer cells *in vivo* and *in vitro*. Furthermore, for OvCa cells, PL-PTX modulated the ERK/Akt pathway and induced apoptosis [145]. Additionally, co-encapsulation of a chemotherapeutic drug (doxorubicin) and a P-gp inhibitor (verapamil) into liposomes conjugated with transferrin showed better responses in terms of high loading efficiencies, selective targeting, high cytotoxicity, and reversal of drug resistance [86].

#### 5.2.3. Micelles

Micelles (size: 10–100 nm) facilitate the penetrability and endocytosis of OvCa cells and impede the targeting of normal cells [156]. Micelles can also deliver water-insoluble chemotherapeutic drugs and inhibit drug resistance via the EPR effect, active internalization, endosomal-triggered release, and drug escape [155,157]. Moreover, micelles accumulate in poorly vascularized tumors, enhance EPR, and increase the half-lives of chemotherapeutic drugs [158]. To test this theory, Xiao et al. [147] investigated if fisetin micelles increase the inhibition of tumor cell growth. A xenograft model of SKOV-3 cells was established. After 21 days of treatment, fisetin micelle treatment led to 70.7% inhibition compared with free fisetin’s 53.6%. Thus, fisetin micelles are more powerful than free fisetin for inhibiting SKOV-3 cells [147].

#### 5.2.4. Mesoporous Silica Nanoparticles

Surface modifications have been explored to facilitate drug loading capabilities. Mesoporous silica nanoparticles (MSNs) have high drug loading efficiency due to their high porous volume and surface area properties. In cancer cells, MSNs have multi-functionalities for targeted and controlled delivery, which allows for enhanced cellular uptake and the delivery of therapeutics at cellular levels [155]. Therefore, because of their advanced pharmacokinetics and treatment efficiency, MSNs are believed to be excellent vehicles for drug delivery [134]. This is a strategy used to treat cancer, mainly aiming to achieve a synergistic effect of a combined drug that can induce the death of tumor cells [159]. In this context, Choi et al. [148] used anti-apoptotic Bcl-2 gene-targeting siRNA (Bcl-2 siRNA) and Ca^2+-^glued it onto bare MSNs, which were then co-loaded with the anti-cancer agent doxorubicin (DOX) to construct siRNA/DOX@Ca^2+^ MSNs. These MSNs were developed to evaluate the synergistic potential of an siRNA drug combination in SKOV-3 cells. These cells showed further compromised viability, showing that Bcl-2 silencing by co-delivered siRNA sensitized cancer cells to DOX-induced apoptosis. Furthermore, for mice, the therapeutic performance of co-delivered siRNA/DOX@Ca^2+^ MSNs significantly increased tumor suppression more than DOX@MSNs and siRNA@Ca^2+^ MSNs, causing the transfected cancer cells to become sensitized to the apoptotic action of co-delivered DOX by Bcl-2 silencing [148].

#### 5.2.5. Dendrimers

Dendrimers are 3D, hyper-branched, and globular nanoparticles. They can be engineered with sizes of 1–15 nm. These nanoparticles have distinctive features, including a low polydispersity index and high-water solubility, biocompatibility, polyvalency, and molecular weights. They can be used to encapsulate both hydrophilic and hydrophobic drugs [86,160]. For the treatment of OvCa cells, Cai et al. (2015) [149] designed a linear–dendritic telodendrimer micelle (TM) nanocarrier to analyze the synergistic effects of delivering two chemotherapeutic agents, cisplatin and PTX, which are hydrophilic and hydrophobic drugs, respectively. The encapsulation of both agents exhibited higher cytotoxicity toward SKOV-3 OvCa cells, demonstrating a synergistic effect. There was greater cytotoxicity for the combination of a 2:1 ratio of cisplatin and PTX in the co-loading formulation at 50% cell killing compared with other treatments (PTX, cisplatin, TM (PTX), and TM (cisplatin)), indicating a synergistic effect on resistant SKOV-3 cells [149]. Overall, since dendrimers are extraordinary drug delivery nanocarriers, research endeavors have been dedicated to evading cytotoxicity and promoting translation into clinical uses [161].

#### 5.2.6. RNA Interference Therapy

Since gene silencing by RNA interference (RNAi) was discovered, many studies have included small interfering RNAs (siRNAs), short hairpin RNAs, or antisense oligodeoxynucleotides as therapeutic options to treat cancer [162]. The constructed siRNA-targeted MDR genes can silence P-gp or MDR1, MRP1, Bcl2, and BCRP and overcome drug resistance. However, to date, the therapeutic efficiency of these RNA interference strategies has not been consistently satisfactory [86]. Therefore, the idea of encapsulating siRNAs into nanoparticles can be effective due to their capacity to avoid rapid degradation of siRNA molecules, its limited exposure to normal cells, its increased cellular targeting, and increased uptake [163]. Yang et al. [150] focused on the MDR gene product P-gp. The encapsulated ABCB1 siRNA hyaluronic acid (HA) nanoparticles targeted cancer cells overexpressing surface protein CD44 [150]. An *in vitro* study was conducted to determine whether the delivered MDR1 siRNA was released from the nanoparticles and retained its functional activity of knocking down the expression of MDR1. For OVCAR8TR PTX-resistant OvCa cells, HA nanoparticle delivery downregulated the P-gp levels, indicating that HA nanoparticles could be used as a therapeutic to re-sensitize cells to PTX [164]. However, to support the biological significance of the in vitro findings and evaluate the antitumor efficacy of HA-PEI/HAPEG/MDR1 siRNA nanoparticles in mice, OVAR8TR were grown as xenografts in nude mice. The results revealed that treatment with HA-PEI/HA-PEG/MDR1 siRNA nanoparticles followed by PTX treatment produced a significant inhibitory effect on the growth of resistant tumors compared with the control groups [150]. Co-delivery of siRNA and chemotherapeutic drugs improves the efficiency of chemotherapy by inducing apoptosis and preventing the expression of anti-apoptotic proteins such as Bcl-2 or survivin [165,166,167,168].

#### 5.2.7. Planetary Ball-Milled Nanoparticles

**Figure 2:**
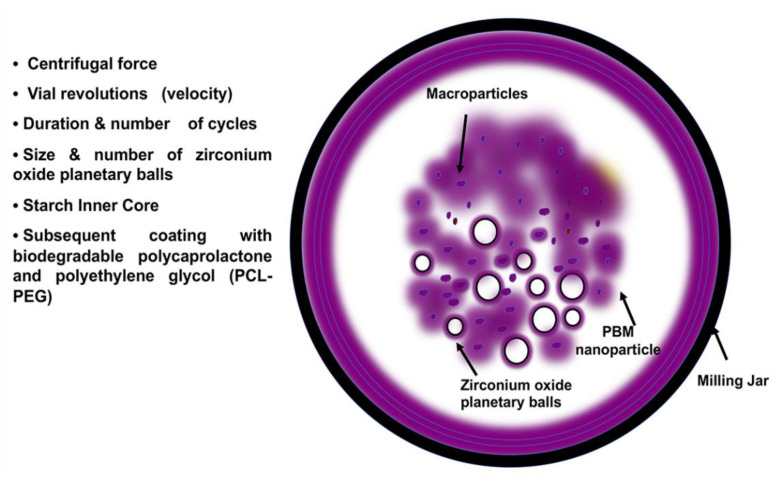
PBM nanoparticle formulation. PBM requires a milling jar, which holds various sizes of heat-absorbent zirconium oxide planetary milling balls. By controlling the size and number of balls, milling cycles, grinding speed, centrifugal force, and planetary jar velocity, PBM can engineer NP size ranges from 5 nm to 60 μm. The arrows indicate the milling jar, PBM nanoparticles containing starch inner cores and encapsulated drugs, zirconium oxide balls, and the macroparticles.

Although other types of nanoparticles are used for drug delivery and targeted therapies, in recent years, planetary ball-milled (PBM) nanoparticles (NPs) have been reported as being more innovative than others. The major issues for the other drug delivery methods are poor aqueous solubility, bioavailability, and absorption [169]. However, PBM nanoparticles are innovative since they are scalable and easy to produce. They have a starch inner core coated with biodegradable copolymers (PCL and polyethylene glycol (PCL-PEG)) milled into a spherical shape and a uniform particle size. Furthermore, Singh et al. (2018) [151] reported that PBM-NPs have 100% loading efficiency for drugs, whether hydrophobic or hydrophilic, and have a control surface logP (metric for hydrophobic and hydrophilic distribution) for systemic, oral, or cutaneous delivery. In PBM techniques, heat-absorbent zirconium oxide planetary milling balls and jars are used to mill the particles that rotate around a shared axis of the chamber wheel. In general, for efficient delivery to the target tumor cells, a round shape and a size <100 nm for the NPs are more favorable [170]. By controlling the size and number of zirconium oxide balls, milling cycles, grinding speed, centrifugal force, and planetary jar velocity, PBM can engineer the NP size range from 5 nm to 60 μm [151,169,171] (Figure 2). Surface polymers can be modified during PBM nano-formulations to deliver encapsulated agents by conjugating targeting molecules such as antibodies, folate, and nucleic acid aptamers [120,171,172] For folate receptor-based targeted therapy, Singh et al. (2018) [151] fabricated PBM-NPs with folic acid on the surface. They encapsulated it with the natural compound RES and docetaxel (DTX) to treat prostate cancer cells. In this study, PBM-NPs coated by folic acid suppressed NF-kB p65, which is involved in inflammation and promotes apoptosis. Furthermore, they showed that PBM-NPs reverse the ABC transporter markers in DTX-resistant PCa cells, limiting the MDR phenotype of the cancer cells. Recent studies also showed that PBM-NPs can target hedgehog signaling pathways in DTX-resistant cells and reverse MDR [148]. Altogether, PBM-NPs have a high potential to encapsulate drugs and are safer and more efficient in the selective targeting of cancer cells. If applied to OvCa cells, they may provide effective therapy for cancers with chemoresistance.

## 6. Conclusions

For cancer therapy, drug resistance has been an enduring problem. Standard cancer treatments frequently lead to MDR and often do not produce cures. However, new strategies can take cancer therapy to higher levels. We have reviewed some of these new strategies, including CAR-T cell therapy, PARPi therapy, natural products, nanoparticles, and RNA interference therapy.

Plants remain a promising pool of drug discovery scaffolds. Natural compounds have various targets, including MDR in cancer cells, have high specificity, are safer, and allow for treatment that has anti-cancer potential with low toxicity. However, rapid metabolism, low bioavailability, and inadequate drug delivery are limiting factors. To overcome these challenges, delivering these compounds in nanocarriers will improve their translation. Nanoparticles and RNA interference therapies allow for targeted and rational treatments with impressive effects. In this context, we [151] have developed PBM engineering technology which allows for the nanoparticle-based drug delivery of hydrophilic and hydrophobic drugs with high loading efficiency, which was previously considered inaccessible. Thus, the idea of combining natural products with chemotherapeutics and encapsulating them in nanocarriers presents a new therapeutic strategy for overcoming drug resistance in cancer cells.

## Figures and Tables

**Table 1:** Mechanisms of action of natural products against OvCa cells.

Natural Product	Cell Type	Mechanism of Action	Reference
Piperine	OVCAR-3 cells	Induces G_2_/M phase cell cycle arrest and caspase activation and inhibits cell migration and the PI3K/Akt/GSK3β signaling pathway	[91]
Flavonoid	PA-1 cells	Decreases viability; induces apoptosis; decreases Bcl-2 and Bcl-xL; and increases caspase-3, caspase-9, Bid, Bad, Bax, and cytochrome c	[92]
Curcumin	Cisplatin-resistant OvCa cells	Induces G_2_/M cell-cycle arrest and increases apoptosis and phosphorylation of p53	[93]
RES	OVCAR-3 cells	Induces ROS generation and apoptosis and activates the autophagy pathway	[94]
Thymoquinone	SKOV-3 cells	Induces apoptosis by decreasing expression of Bcl-2 and increasing expression of Bax	[95]

**Table 2:** Nanocarrier drug therapeutics to treat OvCa.

Nanoparticle Carrier	Therapeutic(s)	Results	Reference
Polymeric nanoparticle	PTX + carboplatin	Increased potency in cells	[143]
Solid lipid nanoparticle	PTX	Cytotoxicity and parenteral routes of administration	[144]
Liposome	PTX	Increased expression of Akt, ERK, and caspase 3/9	[145]
Liposome	PTX + P-gp inhibitor	High loading efficiency, high cytotoxicity, selective targeting, and reversal of P-gp-mediated MDR	[146]
Micelle	Fisetin	Increased cytotoxicity and inhibition of tumor growth	[147]
Mesoporous silica nanoparticles	Bcl-2 siRNA + Doxorubicin	Induced cell death, tumor suppression, and decreased cell viability	[148]
Telodendrimer	PTX + cisplatin	High cytotoxicity and potent synergistic effect of combined nanotherapy	[149]
RNA interference therapy	Hyaluronic acid nanoparticles with siRNA	Suppressed P-gp levels	[150]
PBM nanoparticles	RES + DTX + folic acid	Suppressed NF-kB p65 and reversal of the ABC-transporter markers	[151]
